# Impact of Wind Gust on High-Speed Characteristics of Polarization Mode Dispersion in Optical Power Ground Wire Cables

**DOI:** 10.3390/s20247110

**Published:** 2020-12-11

**Authors:** Jozef Dubovan, Jan Litvik, Daniel Benedikovic, Jarmila Mullerova, Ivan Glesk, Andrej Veselovsky, Milan Dado

**Affiliations:** 1 Faculty of Electrical Engineering and Information Technologies, University of Zilina, 01026 Zilina, Slovakia; jan.litvik@uniza.sk (J.L.); mullerova@lm.uniza.sk (J.M.); milan.dado@uniza.sk (M.D.); 2 Department of Electronic and Electrical Engineering, University of Strathclyde, Glasgow G11XQ, UK; ivan.glesk@strath.ac.uk; 3 Energotel, a.s., 82108 Bratislava, Slovakia; veselovsky.andrej@energotel.sk

**Keywords:** optical fiber, polarization, differential group delay, principal state of polarization, eye diagram, sensing

## Abstract

Polarization mode dispersion is recognized as a key factor limiting optical transmission systems, particularly those fiber links that run at bit rates beyond 10 Gbps. In-line test and characterization of polarization mode dispersion are thus of critical importance to evaluate the quality of installed optical fibers that are in use for high-speed signal traffics. However, polarization-based effects in optical fibers are stochastic and quite sensitive to a range of environmental changes, including optical cable movements. This, in turn, gives rise to undesired variations in light polarization that adversely impair the quality of the signal transmission in the link. In this work, we elaborate on experimental testing and theoretical analysis to asses changes of polarization mode dispersion in optical fibers that are caused by environmental variations, here wind gusts in particular. The study was performed on commercially harnessed optical fibers installed within optical power ground wire cables, taking into account different weather conditions. More specifically, we showed that changes caused by wind gusts significantly influence the differential group delay and the principal state of polarization in those optical fibers. For this, we experimentally measured a number of parameters to characterize light polarization properties. Measurements were carried out on C-band operated fiber-optic link formed by 111-km-long power ground wire cables and 88 spectral channels, with a test time step of 1 min during 12 consecutive days. Variations in differential group delay allowed for sensitive testing of environmental changes with measured maxims up to 10 ps under the worst wind conditions. Moreover, measured parameters were used in a numerical model to assess the quality of transmitted high-bit-rate optical signals as a function of wind conditions. The analysis revealed a negligible impact of wind on a 10 Gbps transmission, while substantial influence was noticed for higher bit rates up to 100 Gbps. These results show promises for efficient sensing of environmental changes and subsequent monitoring of the quality of recently used fiber-optic link infrastructures.

## 1. Introduction

Deployment of photonic systems in many applications has shown a rapid progress in the last decades. Nowadays, optical transmission systems can reach transmission capacity well-beyond 1 Pbps over a single-mode optical fiber [1], with an ever-growing number of newly installed optical cables for many rising areas. This mostly includes short-reach data communications [2], medium-distance metropolitan and access networks [3,4,5], and long-haul continental and trans-continental transmissions [6,7].

Apart from the successful fiber applications in communication and signal transmission, optical fibers can also be designed for sensing and monitoring [8]. Here, the target areas are found on high-way roads, bridges, railways, or power cable lines, to name a few, to avoid weight and thermal overload or fire-related issues [9,10]. Such approaches encompass photonic sensing of specific physical parameters as diverse as temperature, stress strain, or even random environmental changes, which may include temperature, rain, sun, snow, or wind [8,10]. For applications in sensing and monitoring, both single-mode and multi-mode optical fibers are typically employed [8,11]. However, multi-mode optical fibers are not the optimal solution for high-speed optical links compared to their single-mode counterparts. Multi-mode optical fibers suffer from a strong inter-modal interference between excited fiber modes. This results in modal dispersion [12], which is a detrimental effect. Recently, the price difference between single-mode and multi-mode fibers is negligible. To get rid of such problems, single-mode optical fibers are thus preferred for high-speed signal transmission over short and long distances. Besides that, single-mode fibers can provide simultaneous functionality to be exploited as efficient in-line sensors or in-built monitors within the optical transmission links, without the constraints of multi-mode optical fibers.

In single-mode optical fibers, we can distinguish several degradation effects that have a significant impact on transmitted optical signals. Generally, degradation mechanisms can be categorized as deterministic and stochastic, respectively. While the former effects, including attenuation, chromatic dispersion and first-order Kerr nonlinearities, are more or less predictable and can be well-controlled through a range of established compensation schemes, the latter exhibit a non-deterministic nature, which substantially complicates their control [12,13]. Polarization mode dispersion (PMD) is certainly one of the most critical effects with a stochastic nature for fiber-optic transmission systems. The effect of PMD on transmitted optical signals is typically negligible for conventional bit rates that do not exceed 10 Gbps. The PMD origin stems from the random optical birefringence. The random birefringence is dominantly caused by non ideal fiber circularity, coupled with additional changes in vibrations and temperature or bending and twisting. This randomness yields changes in local fiber birefringence axes, which essentially re-defines the light polarization state of output optical signals compared to its initial state. As a consequence, the PMD causes an undesired time delay. This delay leads to temporal spreading of optical pulses that propagate via birefringent optical medium, here optical fiber [14,15]. In other words, different parts of the same optical signal locally travel with different group velocities. This induces a significant time-domain overlap between preceding and consecutive optical pulses, and thus limiting the transmission system bandwidth and attainable system speed. From a system-level perspective, a maximum tolerable delay is up to 10% from the bit length of the transmitted optical pulses [16]. Direct experimental assessment of polarization-based parameters in the fiber-optics links is not easy as both internal and external factors typically come into play. Yet, it is quite useful and equally important for the state-of-the-art optical fiber links to characterize the PMD parameters and their statistics, especially in case of external environmental-based changes, which are indeed challenging to control [15,17,18,19,20,21]. At this point, it is also worth mentioning that there exists a range of possibilities to mitigate the detrimental impact of PMD on high-speed fiber-optic transmission systems. Over years of intense research in the field, several promising and state-of-the-art monitoring approaches and PMD mitigating schemes have been proposed, tested, and deployed into fiber-optic links to get rid of these stochastic impairments that adversely affect the transmission system performances [22,23,24,25,26,27,28,29,30,31,32,33]. This may include the combined deterministic differential group delay (DGD) and polarization maintaining fiber (PMF) methods [25], phase diversity detection method [26], utilizing optical and electrical equalizers [27], two-stage [28] or three-stage [29] PMD compensators, optical compensator based on particle swarm and cross-tracking algorithms [30], using birefringence crystals [31], or chirped fiber Bragg gratings (FBGs) [32], or exploiting automatic compensation concepts [33], among others.

In this work, we report on rigorous measurements of polarization mode dispersion in optical fibers that are influenced by environmental changes—in this case, by the wind gusts. In particular, we show how the wind gust impairs polarization characteristics of commercial optical fibers installed within power wire cables, with a direct link to high-speed signal transmissions via eye diagram inspection.

## 2. Theory, Modeling, and Experiments

### 2.1. Polarization Properties of Optical Fibers: Fundamentals

**Figure 1:**
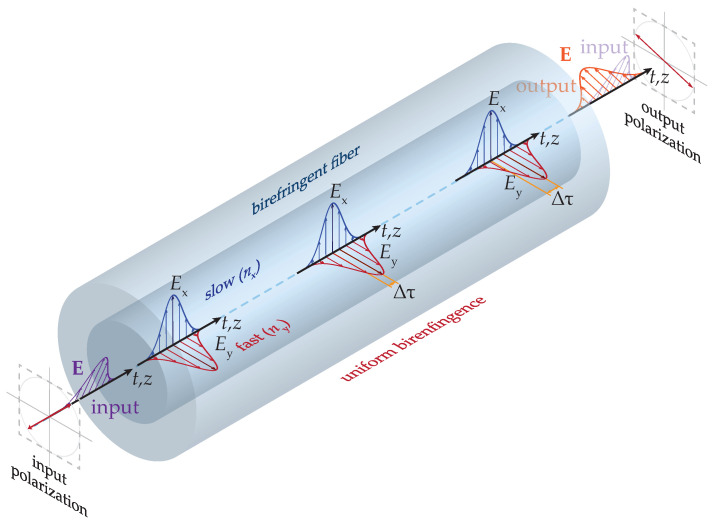
Schematics of the uniform birefringence and its influence on the polarization state of orthogonal modes propagating along the optical fiber.

The refractive index of anisotropic materials depends on light polarization, i.e., different polarized optical waves propagate with different propagation constants. In turn, this yields different phase shifts between them. This effect directly relates to the fiber birefringence. Intrinsic fiber birefringence is typically an unwanted side effect. The intrinsic fiber birefringence arises from the imperfect fiber manufacturing, such as core ellipticity, bending, twisting, or even material impurities and inhomogeneities [34]. As a result, these rather random imperfections cause residual local stress between the fiber core and fiber cladding in each fiber segment. Generally, higher stress induces larger birefringence of the optical fiber and vice versa. This also leads to the different thermal and mechanical properties of conventional silica-based fibers and makes fiber birefringence sensitive to a range of external parameters such as temperature, stress and strain. The birefringence sensitivity then originates from the elasto-optic effect and fiber compressibility [35,36]. As a consequence, polarization characteristics of optical fibers are influenced as well, via a change in a propagation time of two orthogonally polarized modes that run through the fiber with different group velocities. The propagation of two orthogonal modes in the optical fiber is schematically shown in Figure 1. Resulting time changes are conventionally represented by differential group delay (denoted here as DGD or Δτ), and a principal state of polarization (PSP). In situations when optical signal is transmitted via optical fiber, this leads to the effect of PMD with a strong stochastic nature [34,37].

Both internal and external factors can influence the fiber birefringence, and thus both substantially impact the PMD. Internal issues relate mostly to imperfections of the fabrication process. Their control is dominantly driven by the quality of manufacturing, post-fabrication treatment, or even a fiber arrangement within complex optical cables [38]. On the other hand, external factors, especially changes in environmental conditions (rain, wind gust, snow, heat, or temperature variations), mechanical stress and pressures, or even optical fiber/optical cable aging, are much more essential for digital transmission links run with conventional single-mode optical fibers [18,19,20,21]. These different factors, once combined together, typically have a dramatic effect on the intrinsic PMD characteristics, and thus on the performance of fiber-optics systems. The impact of these external changes is recognized to be particularly critical, once the fiber-optic links transmit high-speed optical signals, i.e., fiber-optics transmission systems operating at data rates of 10 Gbps and higher [16]. However, the control of external factors is far from being trivial and easy to realize. Therefore, there is a strong quest to develop and elaborate on strategies for a proper monitoring of these stochastic changes, and subsequently to evaluate their impact on the transmission of high-speed signals within used optical fibers and fiber-based optical links [39].

### 2.2. PMD Modeling and Link Quality Assessment

**Figure 2:**
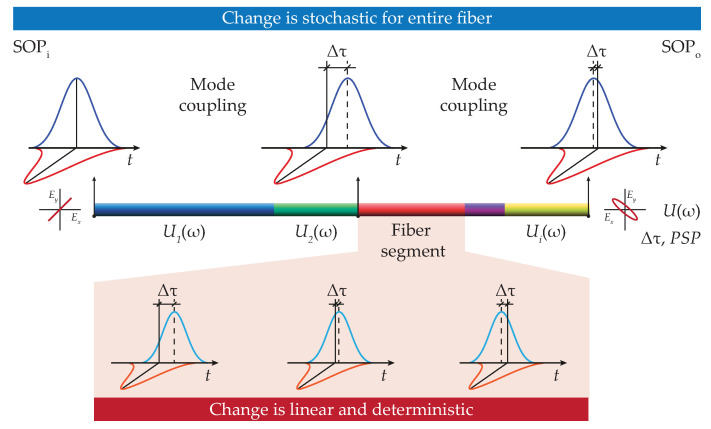
Schematic of the in-house numerical model to study polarization mode dispersion (PMD)-impaired optical pulse propagation in the optical fiber. The light polarization state at the fiber output is completely described by the input state of polarization and propagation of optical pulse through the optical fiber with a randomly varied parameters. Here, each fiber segment (different colored sections along the light propagation direction) is represented with different light polarization properties, i.e., by random birefringence. SOP_*i*_ denotes the initial state of light polarization, SOP_*o*_ is the state of light polarization at the fiber output, $U_{i}\left(\omega \right)$ is the individual Jones matrix, PSP is the principal state of polarization, and Δτ is the differential group delay (DGD). The later parameters used in the in-house model are fully retrieved from the experimental measurements.

In general, any type of external changes give rise to birefringence, in otherwise low birefringent optical fibers. External changes thus enhance the impact of PMD on transmitted optical signals. This way, PMD expresses the dependence of the group velocity and the polarization state of optical signals. Two parameters that are used to fully describe polarization state and PMD are thus PSP and DGD, respectively. In the case of conventional optical fibers designed to transmit optical signals over long (telecommunication) distances, the birefringence distribution is assumed to be homogeneous [40], while in the case of optical fibers utilized for sensing and monitoring, this distribution is typically inhomogeneous [41]. Both parameters, PSP and DGD, together with the input state of polarization (SOP), form the fundamental layout to quantitatively describe polarization-induced fluctuations in optical fibers [42]. Accordingly, these fundamentals are exploited in the in-house numerical model, schematically shown in Figure 2.

For the numerical model, optical fiber is fully described as a sequence of short segments. Individual fiber pieces have different characteristics of the birefringence, characterized by its strength and orientation, respectively. This approach includes a vectorial-based description of the electrical field components, rather than much simpler SOP-averaged analytical methods [16]. Generally, the change of the input state of polarization (SOP_*i*_) is given by the propagation of optical pulse through an optical fiber. Here, the electrical field of the propagating light is characterized by Jones matrix, denoted as $U_{i}\left(\omega \right)$, that corresponds to the particular fiber segment. In the short fiber segments, the birefringence is uniform and the induced change is being linear. In contrast, in long optical fibers, the birefringence is random and the state of polarization at the output (SOP_*o*_) is random as well due to the inherent mode coupling that occurs between two orthogonal light components [34]. It is worth noting that the length of each polarization segment is required to be much shorter compared to the total fiber length. This way, it is assured that the simulation model takes into account a sufficient number of individual fiber segments with randomly varied birefringence.

As illustrated in Figure 2, total optical fiber length is divided into a chain of multiple short-length elements with different degree of birefringence. The in-house model for PMD-impaired optical pulse propagation originates from a vectorial-like Jones formalism for a single fiber segment. In particular, the Jones matrix formalism is described as follows [43]:

(1) $$U\left(\omega \right)=U_{i}\left(\omega \right)U_{i-1}\left(\omega \right)\ldots U_{2}\left(\omega \right)U_{1}\left(\omega \right)=\prod_{i}U_{i}\left(\omega \right),$$

where *i* is the number of total birefringence elements and $U_{i}\left(\omega \right)$ is the particular Jones matrix that relates to the *i*-th element. The particular Jones matrix is thus defined by [15,43]

(2) $$U_{i}\left(\omega \right)=R_{i}\left(\omega \right)\left(\begin{array}{cc} N_{x_{i}} & 0\\ 0 & N_{y_{i}} \end{array}\right)R_{i}\left(-\omega \right)=R_{i}\left(\theta_{i},\varphi_{i}\right)\left(\begin{array}{cc} e^{-j\omega \frac{\Delta \tau_{i}}{2}} & 0\\ 0 & e^{-j\omega \frac{\Delta \tau_{i}}{2}} \end{array}\right)R_{i}\left(-\theta_{i},-\varphi_{i}\right),$$

where $\Delta \tau_{i}$ is the DGD of *i*-th birefringence element and $R(\theta_{i},\varphi_{i})$ is the rotation matrix. Rotation angles $\theta_{i}$ and $\varphi_{i}$ are random variables, typically positioned in the range of −∞ to ∞. They also have an uniform distribution, which is calculated from PSP values.

Then, the optical field at the fiber output for two orthogonal pulse components can be described as follows [43]:

(3) $$E_{out}^{total}={\left(\begin{array}{c} E_{x}\\ E_{y} \end{array}\right)}_{\mathrm{out}}=U\left(\omega \right)E_{in}^{total}=U\left(\omega \right){\left(\begin{array}{c} E_{x}\\ E_{y} \end{array}\right)}_{\mathrm{in}},$$

where $E_{x}$ and $E_{y}$ are input and output electrical fields along the transversal *x* and *y* directions, respectively. The output optical field $E_{out}^{total}$, can be calculated based on input optical field $E_{in}^{total}$, DGD and PSP. It is obvious that the whole problem of the PMD-impaired optical pulse propagation is numerically solved in the frequency domain. Solving the PMD-impaired optical pulse propagation in the frequency domain offers many advantages compared to the pure time-domain pulse propagation methods [16]. In order to retrieve the distorted optical signal back in the time domain, we apply the inverse fast Fourier transformation (IFFT) algorithm as follows [44]:

(4) $$s\left(t\right)={\left|F^{-1}\left\{E_{x,out}\right\}\right|}^{2}+{\left|F^{-1}\left\{E_{y,out}\right\}\right|}^{2}.$$

This model is implemented iteratively, with a segment-by-segment approach, over a total length of the optical fiber.

In optical communication systems, the time delay between two orthogonally polarized modes is defined as follows [12,45,46]:

(5) $$\Delta \tau =D_{PMD}\sqrt{L}\;\;\left[s\right],$$

where Δτ is the average DGD, *L* is the fiber length and $D_{PMD}$ is the PMD fiber coefficient. Here, the random and continual changes in DGD in respect to the optical fiber length represents the characteristics for a random walk of problem, i.e., random delay. Thus, average DGD scales proportionally with the square root of the optical fiber length. This way, the $D_{PMD}$ parameter of the optical fiber actually determines the amount of average DGD variations over $\sqrt{L}$.

As mentioned above, the PMD causes a time delay between propagating modes, as well as changing the energy distribution between two polarization components. From a system-level point of view, this time delay yields a temporal spreading of transmitted optical pulses, which then start to overlap with each other. As a consequence, this results in the temporal-domain effect of inter-symbol interference (ISI). The effect of ISI limits the transmission system performance, especially in terms of available bandwidth and attainable speeds. For a reliable performance of the fiber-optic transmission system, the time spreading tolerance up to 10% of the bit length ($T_{B}$) is accepted for a 1 dB penalty drop [16]. In a case of intensity modulated direct detection (IM-DD) systems with on-off keying (OOK) modulation and non-return-to-zero (NRZ) coding, this tolerance can be expressed as follows:

(6) $$\Delta \tau_{max}=\;\frac{T_{B}}{10};\;T_{B}=\frac{1}{R_{B}},$$

where $\Delta \tau_{max}$ is the maximum tolerable differential group delay, $T_{B}$ is the length of the transmitted bits, and $R_{B}$ is the transmission bit rate.

Increasing data rates well over 10 Gbps, the PMD becomes the limiting factor in high-speed optical communication systems. Advanced processes in optical fiber manufacturing are foreseen to be vital knobs for reducing the PMD impact on high-speed signal traffics [47]. Alternatively, a myriad of PMD compensation techniques can be exploited as well to mitigate the influence of external variations, and thus reduce the impact of the PMD on optical transmission systems [22,23,24,25,26,27,28,29,30,31,32,33].

In general, the PMD is well-described by its magnitude and orientation. There are various techniques and parameters that can be directly implemented, all of them indicating the impact of PMD. This includes DGD histograms, eye diagram openings, degree of polarization at the various frequencies and state of light polarization [18,19,20,21,47].

The quality of the transmitted high-speed optical signals is evaluated via eye diagram. Optical NRZ signal with a specific bit rate is degraded by the PMD based on Equation (4). Then, the eye diagram opening, EO, is defined as follows [48,49]:

(7) $$EO=s_{1,min}-s_{0,max},$$

where $s_{1,min}$ is the minimum value of “1” bit and $s_{0,max}$ is the maximum value of “0” bit.

The eye opening penalty (EOP), i.e., eye diagram closure, is used to assess the quality of the link. Here, we note that to demonstrate an impact of pure PMD on high-speed optical signals, we omitted the noise characteristics of the transmitter and receiver in the numerical model and its performance evaluation method. The inputs for our in-house model come exclusively from experimental data, and not from simulation itself.

The EOP is defined as [49]

(8) EOP=−10logEO[dB].

The normalized eye diagram openings depend on DGD and PSP and ranges from 0 to 1. The 2 dB penalty is defined to maintain a reliable and satisfactory signal transmission in the fiber-optic system [50].

### 2.3. Experimental Test Set-Up

**Figure 3:**
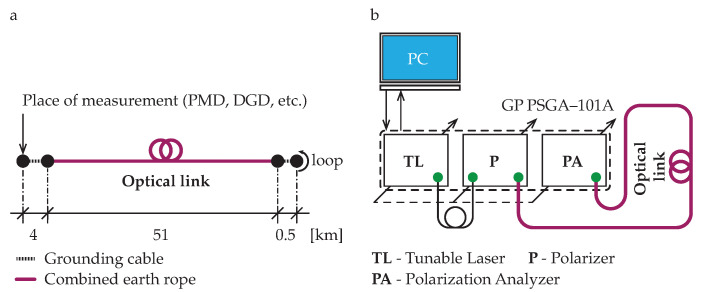
(**a**) Schematics of the back-to-back optical link used in experiments. (**b**) Optical test set-up used to measure parameters of the optical link.

Measurements were performed on optical power ground wire cables with a commercial traffic, combining functions of grounding and communications, respectively. The measured optical cable contains single-mode optical fibers according to ITU-T G.652 recommendation [51]. The cable comprises several optical fibers surrounded by layers of steel and aluminum wires. The overall length of the measured optical path was 111 km. At the beginning and ending of the measured link, the cable was installed in the ground for 4 km and 0.5 km, respectively. For the rest of the measured link, optical fibers within cables hung in the air along the distance of 51 km in a back-to-back configuration. The optical link schematics are shown in Figure 3a.

The experimental test set-up is shown in Figure 3b. The set-up consists of PMD analyzer GP PSGA-101A and measured optical link itself. For the whole tests, the Jones matrix eigen analysis (JME) method was exploited [52]. Measurements were carried out simultaneously using available 88 spectral channels in the wavelength range from 1528.97 nm to 1563 nm. The transmission channels were separated with standard channel spacing of 50 GHz according to the ITU-T dense wavelength division multiplexing (DWDM) grid recommendation [53].

Measurements took place in Energotel, a.s. Zilina over a time period of 12 days, ranging from 15 to 26 February 2020 in particular. The total number of measurements was 15,000 with a data acquisition rate of 1 min. Table 1 sums up all parameters in use during PMD measurements. As a result, the following set of optical fiber parameters was extracted from the measurements:

- time of measurement,
- differential group delay (DGD),
- second order differential group delay (SODGD),
- principal state of polarization (PSP),
- polarization dependence loss (PDL).

In the following, only measured values of DGD and PSP are considered for further analysis, as those two parameters form the fundamental basis of the PMD and are also well-positioned within the established numerical model. Monitored changes in measured parameters arise from comparatively longer length of optical fibers hanging in the air, rather than the fiber installed in the ground. The former part of the link represents up to 92% of total link length. For clarity, contribution to the PMD changes in the later link part can be neglected.

## 3. Results and Discussion

**Figure 4:**
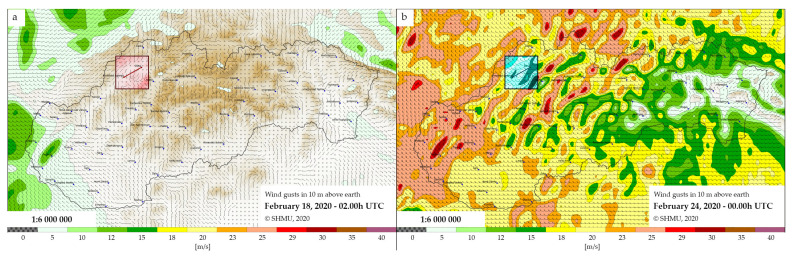
Wind conditions in selected country area. (**a**) A day of weak wind conditions with a wind speed up to 5 m/s. (**b**) A day of strong wind conditions with a wind speed up to 20 m/s. Highlighted squared boxes show the testing area, where the optical power wire cables were located.

Earlier described experimental work performed on the selected optical link is here correlated with an impact of wind gusts. For this, the measured optical link is located in a close proximity of about few kilometers to the hydrometeorological station, where the measurement of environmental conditions such as wind speed and temperature were performed. The information about the weather conditions during testing days is shown in Figure 4, for two different days with comparatively different weather situations. Different wind conditions allow: (i) much better experimental study and (ii) a reasonable correlation between retrieved polarization-based parameters and an actual weather situation. This, in turn, would enable us appropriate evaluation of the optical link quality. The data reported in Figure 4 were modeled using ALADIN simulation tool provided by Slovak Hydrometeorological Institute. It is apparent that the wind situation was better on 18 February 2020 (weak wind conditions, up to 5 m/s) than the wind situation on 24 February 2020 (strong wind conditions, up to 20 m/s). This way, we expect larger values for measured polarization parameters in the latter case rather than in the former situation. As a consequence, the optical link quality will go down as well, especially for higher data rates.

It is also important to mention that the local changes in the weather conditions, i.e., wind speed variations over a short period of time, can potentially introduce a certain level of uncertainty in the performed measurements. To get rid of these problems, we performed a robust 12-days-long testing with a 60 s data collection rate, as well as the measured data; these were later averaged to alleviate potential uncertainties (see discussion below for more details).

**Figure 5:**
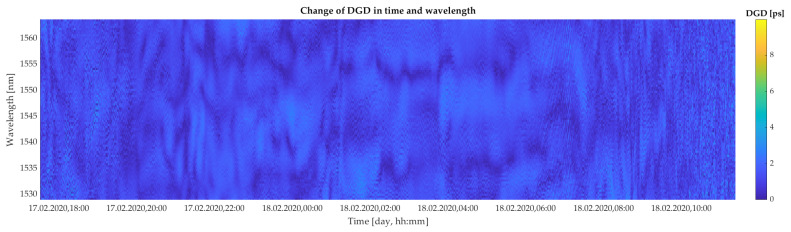
Colormap of the experimentally retrieved values of DGD as a function of 88 spectral channels in bandwidth ranging from 1528.97 nm to 1563 nm and selected time slots under a weak wind condition (wind speed up to 5 m/s).

**Figure 6:**
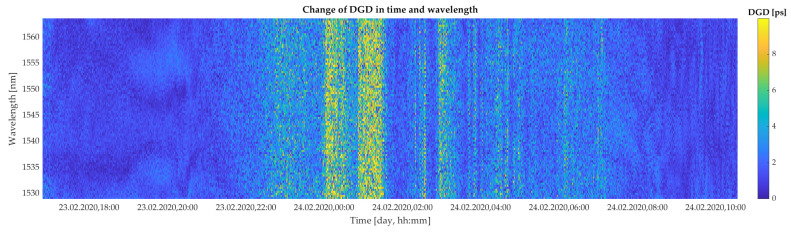
Colormap of the experimentally retrieved values of DGD as a function of 88 spectral channels in bandwidth ranging from 1528.97 nm to 1563 nm and selected time slots under a strong wind condition (wind speed up to 20 m/s).

Figure 5 and Figure 6 show the evolution of the DGD as a function of all 88 spectral channels and specific time intervals of measurements. Results reported in Figure 5 and Figure 6 correspond to the wind situations depicted in Figure 4a,b, respectively. This way, both extremes with weak and strong wind conditions, and thus higher impact of the wind gusts, are considered in our study. The yellow color in Figure 5 and Figure 6 express the highest measured values of DGD. The highest changes in DGD were observed on 24 February 2020, from 00.00 to 02.00 a.m. These data sets contain 88,000 values of DGD.

**Figure 7:**
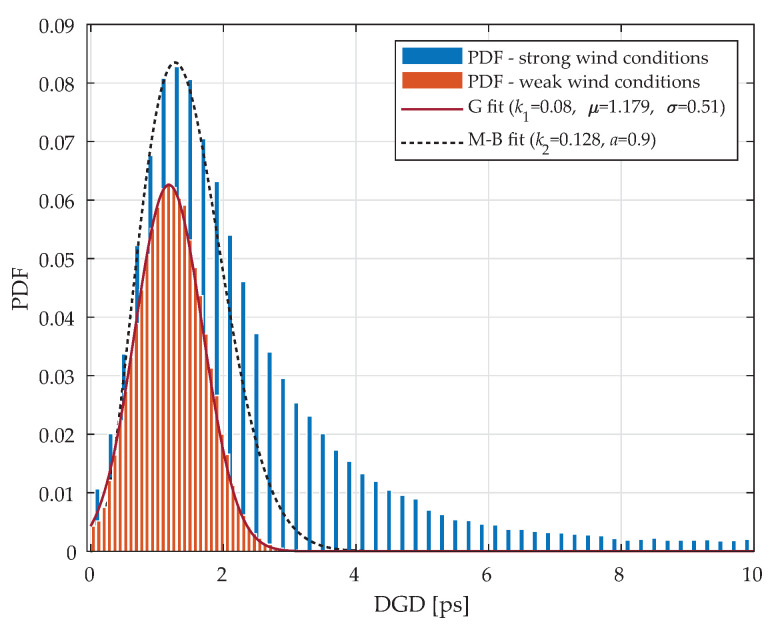
Probability density functions versus DGD retrieved from experimental measurements. The probability density functions are then fitted with different models: Gaussian model for weak wind conditions and Maxwell–Boltzmann model for strong wind conditions.

Figure 7 shows probability density functions (PDF’s) of measured DGDs for all 88 wavelength channels. In case of weak wind conditions (orange histograms), we can see that the resulting PDF resembles a Gaussian-like curve, with an average DGD of 1.17 ps and a maximum of 4 ps. The experimentally retrieved distribution is then fitted as follows:

(9) $$g\left(\Delta \tau \right)=k_{1}\frac{1}{\sigma \sqrt{2\pi }}e^{-\frac{1}{2}{\left(\frac{\Delta \tau -\mu }{\sigma }\right)}^{2}},$$

here, $k_{1}$ is a scaling constant, σ is the standard deviation and μ is the mean value. The corresponding coefficient are listed in the plot. In contrast, in the case of strong wind conditions (blue histograms), the PDF from the retrieved experimental data follows a Maxwell–-Boltzmann distribution [54]. In this case, the average DGD has a slightly higher peak at 1.3 ps and a maximum of DGD was 10 ps. This demonstrates that there is a rather strong relation between the actual weather conditions (weak and strong wind of 5 and 20 m/s, respectively) and the measured values of DGD, although the average DGDs are smaller. In addition, for the latter case, experimental-based PDF was also fitted with a Maxwell–Boltzmann model, that is defined as follows:

(10) $$mb\left(\Delta \tau \right)=k_{2}\sqrt{\frac{2}{\pi }}\frac{\Delta \tau^{2}e^{\frac{-\Delta \tau^{2}}{2a^{2}}}}{a^{3}},$$

here, mb is Maxwell–Boltzmann function, Δτ is DGD, $k_{2}$ is scaling constant, and *a* denotes a distribution parameter. The model fit coefficients are provided in the plot.

The fact that under a stronger wind condition, the DGD distribution follows a known Maxwell–Boltzmann distribution strengthens our findings. In the case of Gaussian-like PDF distribution, the weak wind conditions induced only smaller temporal DGD changes, and DGD values are probably more related to the intrinsic noise, which has a near-Gaussian distribution.

**Figure 8:**
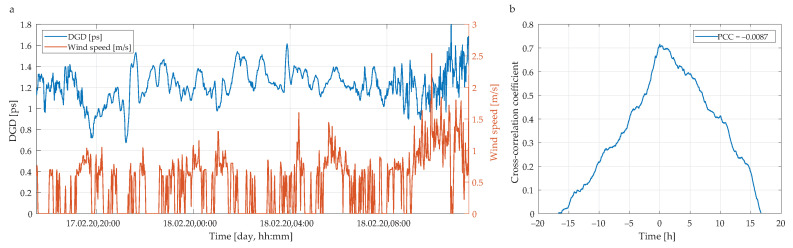
(**a**) Measured changes in average DGD and wind speed versus the measurement time. (**b**) Cross-correlation between measured DGD values and PCC. Weak wind condition (wind speed up to 5 m/s).

For a more complex study, we use the average values of the DGD from all 88 spectral channels rather than the respective single data points. Figure 8a shows the average DGD values (blue line) and wind speeds (red line) as a function of a time. To quantify the correlation between the measured DGD values and the actual wind conditions, we use cross-correlation coefficient defined as follows [55]:

(11) $$X_{\tau_{a},w,coeff}\left(m\right)=\frac{1}{\sqrt{X_{\tau_{a},\tau_{a}}\left(0\right)X_{w,w}\left(0\right)}}X_{\tau_{a},w}\left(m\right),$$

where $\tau_{a}$ is average value of DGD, *w* is wind speed and *X* denotes cross-correlation. The resulting cross-correlation coefficient is shown in Figure 8b.

For this, we obtain no linear correlation, i.e., the Pearson correlation coefficient (PCC) is close to zero. The PCC is defined as follows [56,57]:

(12) $$\rho \left(\tau_{a},w\right)=\frac{\mathrm{cov}\;\left(\tau_{a},w\right)}{\sigma_{\tau_{a}}\sigma_{w}},$$

where cov denotes covariance and σ standard deviation. This result implies that there is only a negligibly small dependence between the actual weather conditions (here, wind speed of 5 m/s) and the average DGD retrieved from the experimental measurements.

**Figure 9:**
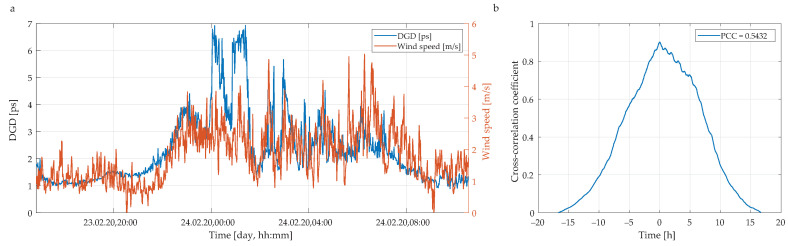
(**a**) Measured changes in average DGD and wind speed versus the measurement time. (**b**) Cross-correlation between measured DGD values and PCC. Strong wind condition (wind speed up to 20 m/s).

In contrary, in case of strong wind conditions, the measured lines of average DGD and wind speed are obviously more correlated. Figure 9a shows the average DGD values (blue line) and wind speeds (red line) as a function of a time. In this case, the cross-correlation coefficient is sharper (see Figure 9b) and yields linear correlation, with a PCC of about 0.54. That indicates a very strong dependence between wind conditions and measured DGD values.

Moreover, according to Figure 8b and Figure 9b, respectively, it can be seen that those signals are not substantially similar in respect to time, i.e., there is zero time-shifted similarity. Yet, a certain level of time symmetry between signals is present in case of strong wind condition. This is caused via similar rising and dropping trends in that function (Figure 9b). A partly curving for correlation function in case of weak wind conditions (Figure 8b) may indicate that linear correlation will be minimal. It also becomes clear that signals for weak wind conditions are not linearly correlated. On the contrary, for a strong wind condition, signals are in robust linear correlation. Accordingly, we may say that for strong wind condition, there may exist a causal relation with DGD changes, and the impact is immediate without time shifting. This is also apparent from Figure 6, that all spectral channels are affected in a very similar fashion.

**Figure 10:**
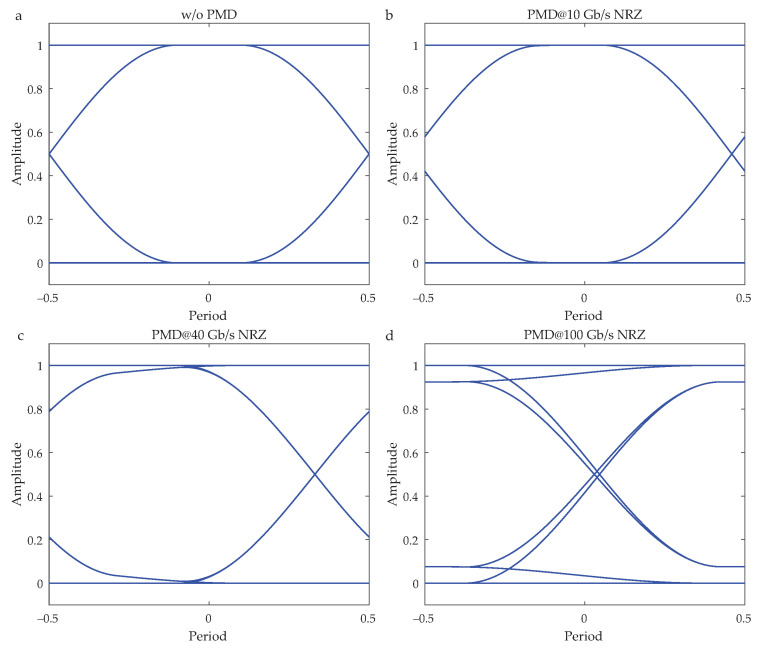
Eye diagrams impacted by PMD under strong wind conditions for different transmission bit rates. (**a**) Reference eye diagram at 10 Gbps without the impact of PMD. Wind-impaired eye diagrams for bit rates of: (**b**) 10 Gbps, (**c**) 40 Gbps, and (**d**) 100 Gbps. To calculate the signal degradation, the following parameters were considered: Δτ=9.493 ps and PSP=[−0.182,0.497,0.849].

The impact of PMD on high-speed optical signals is evaluated via eye diagrams. Figure 10a shows reference eye diagram (without a PMD impact) of an ideal input optical signal in a NRZ format and a data rate of 10 Gbps. The reference eye diagram exhibits a maximal eye opening.

Figure 10b–d show wind-impaired eye diagrams of optical signals with a 64-bit sequence and this for different bit rates of 10, 40, and 100 Gbps. To demonstrate the impact of the PMD, a set of measured parameters (Δτ=9.493 ps and PSP=[−0.182,0.497,0.849]) was selected. These values correspond to the strong wind conditions. As expected, eye diagram stays open and unchanged for a bit rate of 10 Gbps. In contrast, in the case of 40 Gbps and 100 Gbps, calculated eye diagrams start to have an eye closure. This clearly indicates a higher probability of errors for the transmitted data. In the worst case, this could yield an unreliable transmission via optical link.

**Figure 11:**
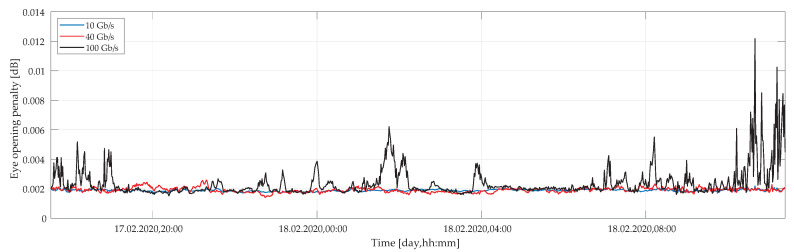
Calculated eye opening penalty as a function of time under weak wind conditions for several transmission bit rates of 10, 40, and 100 Gbps.

Moreover, the eye opening penalty was calculated for each measured set of polarization parameters in time and wavelength. Figure 11 shows average values of EOP under a weak wind conditions as a function of a time for different bit rates of 10 Gbps, 40 Gbps, and 100 Gbps. We may observe only a marginal impact of EOP under such weather circumstances. In the worst case, i.e., for 100 Gbps, the EOP hardly reaches 0.012 dB level, which is good enough to maintain a reliable link operation.

**Figure 12:**
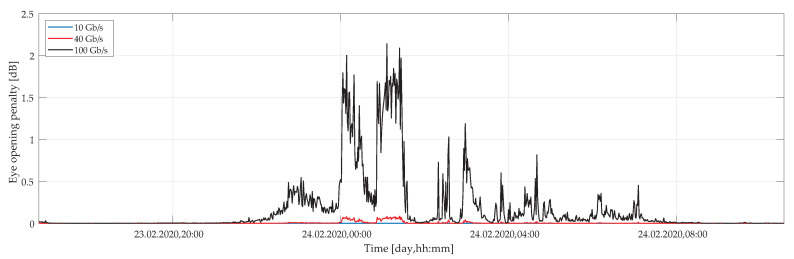
Calculated eye opening penalty as a function of time under strong wind conditions for several transmission bit rates of 10, 40, and 100 Gbps.

Figure 12 shows an average EOP in case of a strong wind as a function of a time for the same range of transmission bit rates. Compared to the previous case (weak wind conditions), we may observe a significant difference. This difference reaches almost three orders of magnitude. This is particularly critical for the 100 Gbps data rate. For this, the EOP is more than 2 dB. This suggests that the quality of the link goes substantially down under such conditions. In turn, this may affect the overall operation of the fiber-optics link, yielding an unreliable signal transmission.

It is also worth mentioning that IM-DD systems, as is the case of this experimental study, have their own limitations, especially in terms of PMD compensation and further system scalability. In contrast, emerging coherent transmission systems working with advanced multi-level modulation formats, such as 16- or 64-quadrature amplitude modulation (16- or 64-QAM), offer much better opportunities for PMD compensation and improved system capacity. The PMD-impaired signal transmission can be almost completely compensated in coherent systems with 16- or 64-QAM signals using advanced techniques of digital signal processing (DSP). As long as the DSP schemes and electrical equalizers are sufficiently robust and complex, the PMD-related link impairments are not critical aspect for high-performing optical transmissions within coherent digital systems [58,59].

Indeed, several appealing schemes and techniques to compensate for a detrimental impact of PMD on IM-DD fiber-optic transmission systems are available to date [22,23,24,25,26,27,28,29,30,31,32,33]. In order to improve the transmission quality of the fiber-optic link under investigation, and thus enable higher operation speeds to be potentially considered in future, attractive compensation approaches to be adopted may include optical compensators with smart and intelligent algorithms [30], chirped FBGs [32], or even automatic-driven compensation concepts [33].

## 4. Conclusions

In summary, we reported on direct measurements of polarization-related parameters in commercial optical fibers installed within power ground wire cables within 111-km-long optical link. In particular, we measured DGD and PSP variations, both being a key for PMD, in respect to the environmental changes, here dominantly caused by wind gusts. We performed robust in-line tests and measurements with 88 spectral channels and a 1-minute time step under different weather conditions across 12 consecutive days. According to the performed experimental tests, we measured differential group delay up to 4 ps and 10 ps for weak and strong wind conditions, respectively. Through the detailed cross-correlation analysis, we showed that the wind with a speed of about 20 m/s can have a substantial impact on polarization parameters, while in the case of a weak wind with a speed up to 5 m/s, that impact is comparatively lower and nearly negligible. In addition, we used measured values in the numerical model to assess the optical link quality. Here, we demonstrated that under weak wind conditions (wind speed up to 5 m/s), the optical link under study was able to handle high-bit rate traffics up to 100 Gbps without a significant penalty on fiber link operation. In contrast, under a strong wind conditions (wind speed up to 20 m/s), a vital link activity could be maintained for 10 Gbps and 40 Gbps signals. Indeed, this sets good prospects for the recently used 10 Gbps Ethernet lines with scaling capability towards 40 Gbps systems. On the other hand, however, we observed a significant penalty for 100 Gbps bit rates for strong wind conditions. This would hinder a reliable link operation at such high bit rates. Our work reveals promises for credible sensing of environmental changes within the fiber-optic links and simultaneous monitoring of the fiber-optic link quality within employed infrastructures. Our finding sets good prospects for scaling installed fiber-optic systems towards much faster transmission links.

## Figures and Tables

**Table 1:** Summary of parameters in use for experimental work.

Parameters	Description
Length of the fiber link	Total: 111 km, where 9 km in the ground and 102 km in the air
Fiber link arrangement	Back-to-back architecture
Fiber type	ITU-T G.652 recommendation (1997)
Number of channels	88
Channel spacing	50 GHz
Wavelength band	Conventional (C) communication window
Wavelength span	1528.97 nm to 1563 nm
Testing period	15 to 25 February 2020
Length of the testing period	12 days
Test equipment	PMD analyzer GP PSGA-101A
Data acquisition rate	60 s

## Abbreviations

The following abbreviations are used in this manuscript:

ALADIN	Aire Limitée Adaptation Dynamique Développement International (French)
DWDM	Dense Wavelength Division Multiplexing
EO	Eye diagram Opening
EOP	Eye Opening Penalty
FBG	Fiber Bragg Grating
Gbps	Gigabit Per Second
IFFT	Inverse Fast Fourier Transformation
IM-DD	Intensity-Modulated Direct Detection
ISI	Inter-Symbol Interference
ITU-T	International Telecommunication Union—Telecommunication Standardization Sector
JME	Jones Matrix Eigen Analysis
NRZ	Non-Return-to-Zero
OOK	On-Off Keying
P	Polarizer
PA	Polarization Analyzer
Pbps	Petabit Per sScond
PCC	Pearson Correlation Coefficient
PDF	Probability Density Function
PDL	PolarizatIon Dependence Loss
PMD	Polarization Mode Dispersion
PMF	Polarization Maintaining Fiber
PSP	Principal State of Polarization
QAM	Quadrature Amplitude Modulation
SHMU	Slovak Hydrometeorological Institute
SODGD	Second Order Differential Group Delay
SOP	State of Polarization
TL	Tunable Laser
